# Psychological Impacts among Older and Younger People Living with HIV/AIDS in Nanning, China

**DOI:** 10.1155/2014/576592

**Published:** 2014-07-14

**Authors:** Hongjie Liu, Xin He, Judith A. Levy, Yongfang Xu, Chunpeng Zang, Xinqin Lin

**Affiliations:** ^1^Department of Epidemiology and Biostatistics, School of Public Health, University of Maryland, College Park, MD 20742, USA; ^2^Health Policy and Administration, School of Public Health, University of Illinois, Chicago, IL 60612, USA; ^3^Nanning Center for Disease Control and Prevention, Nanning 530023, China

## Abstract

*Objectives*. The HIV epidemic has drastically increased among older adults in China, yet little research has examined the psychological impacts among older and younger people living with HIV/AIDS (PLWHAs). This study examined and compared self-efficacy, depression, well-being, and quality of life among older and younger PLWHAs in China. *Method*. A two-stage sampling procedure was used to recruit a final sample of 148 participants. Older adults were defined as age 50 and older. *Result*. Compared to younger PLWHAs aged 18–49 years old, older PLWHAs reported lower levels of well-being (7.6 versus 11.4), higher levels of depression (18.6 versus 15.8), and poorer quality of life. Self-efficacy was similar among older (23.9) and younger (24.6) PLWHAs. A higher level of depression among older PLWHAs was associated with much lower levels of subjective well-being and quality of life (physical health and psychological health). *Conclusion*. The findings suggest that older PLWHAs face psychological problems and mental health challenges beyond those experienced by younger PLWHAs. Intervention programs dedicated to improving mental health and quality of life are greatly needed for HIV infected older adults.

## 1. Introduction

According to a recent report by the Chinese Ministry of Health, the proportion of people age 50 or older living with HIV/AIDS (PLWHAs) among the total reported HIV cases in China increased 11-fold from 1.9% in 2000 to 21.1% in 2011 [1]. In some geographic areas of the country, the HIV prevalence among older adults has rapidly increased. For example, from 2007 to 2011, the proportion of older PLWHAs among the total reported HIV cases increased from 16.5% to 42.7% in Nanning, one of the HIV epicenters in China [2].

The aging of the AIDS epidemic is occurring globally, not just in China. By 2015, about half of PLWHAs in the United States will be 50 years of age or older [3], with comparable trends reported in Sub-Saharan Africa [4] and Australia [5]. As effective antiretroviral therapy (ART) becomes increasingly available throughout parts of the world, many PLWHAs who were infected as young or middle-aged adults now survive into older age. Additionally, growing older does not necessarily prevent these individuals from engaging in risky behaviors and contracting the virus.

Living with HIV/AIDS can be extremely challenging at any age, but older PLWHAs have to contend with the physical declines of an aging body. As both aging and HIV infection can work separately or interactively to reduce human immune response, older PLWHAs are particularly susceptible to AIDS related or non-AIDS related chronic diseases [6]. While ART has led to a marked reduction in the incidence of AIDS-defining illnesses, a variety of HIV associated non-AIDS (HANA) conditions are becoming increasingly commonplace in individuals with long standing HIV infection [7]. These conditions include cardiovascular disease, lung disease, infection-related and non-infection-related cancers, neuropsychiatric disorders, liver cirrhosis, and renal disease. As reported by Negin et al. [8], HIV-infected older adults in South Africa have high rates of chronic diseases, compared with those aged 18–49 years old.

In addition, the interaction of aging processes and AIDS-associated conditions can create considerable psychosocial challenges for older PLWHAs. Social relationships may become strained or disrupted due to HIV's association with stigmatizing behaviors and also through social discrimination [9, 10]. Acquiring and living with HIV can lead to psychological difficulties and reduced mental health, including decreased self-efficacy, depression, impaired well-being, and poor quality of life. Age-related reductions in immune response, impaired physical function, and reduced social support can contribute to or exacerbate existing psychological and mental problems [11]. Compared to their younger counterparts, older PLWHAs may experience greater difficulty coping with HIV-driven psychological challenges and mental health due to the correlates of advancing age.

Despite the rapidly increasing number of older adults living with the virus globally, little research to date has compared psychosocial health of older PLWHAs to younger PLWHAs [9, 12]. Moreover, almost nothing is known about how HIV may affect the psychosocial functioning and the well-being of older PLWHAs in China and other Asian countries. The scant findings that are available for China report that older PLWHAs often worry that disclosing their positive status will lead to social isolation and rejection [13], especially if the source of infection is through paid sex or illicit drug use [14]. Older adults also tend to internalize feelings of worthlessness and self-blame for becoming infected [13].

To understand the psychological challenges that older adults confront living with HIV and also to obtain baseline data for further research, we conducted a study of older PLWHAs in China. Consistent with other HIV research [7], we defined older PLWHAs as men and women at age 50 and older. Younger PLWHAs were defined as 18–49 years old. The objectives of this study were to examine the patterns of psychological challenges faced by older PLWHAs in China and to compare their levels of self-efficacy, depression, psychosocial well-being, and quality of life to younger PLWHAs.

## 2. Methods

The study protocol and consent procedures were reviewed and approved by the Institutional Review Boards of Virginia Commonwealth University and Guangxi Center for Disease Control and Prevention. In accordance with the approved protocol, written informed consent was obtained from all study participants prior to data collection.

### 2.1. Study Sites and Subjects

The cross-sectional study was conducted in Nanning, the capital city of the Guangxi Province. In 2011, Guangxi ranked second among China's 31 provinces for reported number of HIV infections. Although about 30% of all PLWHAs (including both younger and older ones) in Nanning acquired HIV through heterosexual intercourses and 45% through needle-sharing, the dominant HIV transmission mode among older adults was heterosexual (90%) [2].

A two-stage sampling approach was used to recruit eligible subjects [15]. Eligibility criteria included PLWHAs who were at least 18 years old and able to participate in a face-to-face interview. For the first stage, we purposely selected three study sites that provided HIV care and treatment services for the majority of PLWHAs in the city: an infectious disease hospital that was designated to provide care and treatment for PLWHAs, a methadone maintenance treatment (MMT) clinic run by Nanning Center for Disease and Control, and a healthcare center run by PLWHA volunteers. During the second stage, based on a list of PLWHAs in each site, all eligible subjects were invited to participate in the study during the study period. HIV status was confirmed by surveillance data provided by the Nanning Center for Disease Control and Prevention.

### 2.2. Measures

In addition to collecting basic demographic information, we administered four well-validated scales to assess quality of life, general self-efficacy, subjective well-being, and depression. The demographic items and scales initially were drafted in English and then translated into Chinese by three research team members who were fluent in both languages. The Chinese version of the measurement items was then distributed to research team members for further review and word-modified to assure that the measures were appropriate within a Chinese context. The four scales are as follows.

*The Chinese version of the World Health Organization Quality of Life Assessment (WHOQOL-BREF)* was used to measure the level of quality of life [16, 17]. The WHOQOL-BREF consists of 26 items that measure four QOL-domains: physical health (pain, energy, sleep, mobility, activities, medication, and work), psychological health (positive and negative feelings, cognitions, self-esteem, body image, and spirituality), social relationships (personal relationships, social support, and sexual activities), and environmental aspects (safety and security, home environment, finances, health and care, information, leisure, physical environment, and transport). Interviewees responded to these items on a five-point Likert scale. The four-domain scores indicated an individual's perception of quality of life in each particular domain. Domain scores were scaled in a positive direction (i.e., higher scores indicate better quality of life). Cronbach's reliability alpha of the measurement domains was between 0.43 and 0.85 (0.79 in physical health, 0.76 in psychological health, 0.43 in social relations, and 0.85 in environment) in older PLWHAs and 0.64–0.84 in younger PLWHAs (0.83 in physical health, 0.84 in psychological health, 0.64 in social relations, and 0.83 in environment). The domain score was calculated as the mean score of items within each domain.

*The general self-efficacy scale* is a 10-item scale used to measure perceived self-efficacy [18]. Sample items included, “I can always manage to solve difficult problems if I try hard enough” and “I can solve most problems if I invest the necessary effort.” Participants responded on a four-point scale ranging from “not at all true (1)” to “exactly true (4).” Cronbach's alpha was 0.87 in older PLWHAs and 0.84 in younger PLWHAs. A composite score was calculated by totaling the responding value of the 10 items. The composite scores ranged from 1 to 40 with higher scores indicating higher self-efficacy.

*The WHO well-being index* was used to measure the level of subjective well-being [19]. This index consisted of five measurement items on a six-point scale ranging from “at no time (0)” to “all of the time (5).” Sample items included, “I have felt cheerful and in good spirits” and “My daily life has been filled with things that interest me.” Cronbach's alpha was 0.95 in older PLWHAs and 0.92 in younger PLWHAs. A composite score was calculated by totaling the value of the five items. Higher scores represent better well-being.

*Center for Epidemiologic Studies Depression Scale (CES-D)* is a 10-item scale designed to measure depressive symptoms experienced in the past week [20]. Responses ranged from 0 to 3: 0 = rarely or none of the time (less than 1 day); 1 = some or a little of the time (1-2 days); 2 = occasionally or a moderate amount of the time (3-4 days); and 3 = most or all of the time (5–7 days). The CES-D total score was calculated by adding the scores for all 10 items ranging from 0 to 30. Cronbach's alpha was 0.78 in older PLWHAs and 0.82 in younger PLWHAs. Higher scores indicate greater depression.

The above measurement scales have been used successfully in other studies with Chinese populations [16, 18, 21, 22]. In addition, HIV knowledge regarding transmission and prevention of HIV/AIDS was measured by 11 true/false/unsure questions. One point was given for a correct answer, with a possible score ranging from 0 to 11 points.

### 2.3. Data Analysis

Chi-square tests were used to determine statistical differences in demographic variables (i.e., gender, education, and marital status) between older and younger PLWHAs. Pearson's correlation coefficients were calculated to measure bivariate correlations. The comparisons of psychosocial and mental health between older and younger PLWHAs were conducted using Student's *t* tests, given that the normality assumption was not violated; otherwise, a logarithmic transformation was applied to remove skewness. General linear models were used to estimate and compare adjusted means of HIV knowledge, self-efficacy, depression, well-being, and quality of life after accounting for gender, education, marital status, current ART treatment, and self-reported years since HIV diagnosis. SAS Version 9.3 (SAS Inc., Cary, NC) was used to perform all the analyses.

## 3. Results

### 3.1. Demographic Characteristics of the Two Groups

A total of 170 PLWHAs were invited to participate in the study. Of these, 20 PLWHAs declined participation due to unavailability (*n* = 18) or failure to provide information on key measures (*n* = 2), resulting in a total of 148 PLWHAs (31 older PLWHAs and 117 younger PLWHAs). The mean age of older PHWHAs was 58.4 years old (range: 50–80 years old) and 36.0 (range: 20–49) for younger PLWHAs. Across both age categories of PLWHAs, length of time between HIV diagnosis and the interview ranged from 1 month to 13.5 years, with a median of 18 months (12 months among older PLWHAs and 19 months among younger PLWHAs). China's Comprehensive AIDS Response Program (China CARES) is the single largest AIDS project in the world, covering 83.3 million people in 127 program sites in 28 provinces [23]. Under this program, forty-eight percent of older PLWHAs (15/31) and 56% of younger PLWHAs (65/117) received ART. Compared to the younger PLWHAs, older PLWHAs were more likely to be married (87% versus 66%). No statistically significant differences were found for the variables: gender, education, number of years after HIV diagnosis, and being on ART among older and younger PLWHAs (Table 1).

### 3.2. Comparisons of Psychosocial and Mental Health between Older and Younger PLWHA

Using a cut-off score of 10 or more to classify participants as having depressive symptoms [24, 25], the prevalence of depressive symptom was 74.2% (23/31) among older PLWHAs and 48.7% (57/117) in younger PLWHAs (*χ* ^2^ = 6.40, *P* = 0.01). Compared to younger PLWHAs, older PLWHAs reported lower levels of well-being (7.6 versus 11.4) and higher levels of depression (18.6 versus 15.8). There were no significant differences in self-efficacy (23.9 versus 24.6) and HIV knowledge (9.3 versus 10.3) between older and younger PLWHAs, respectively.

Three out of four quality of life domains (physical health, psychological, and social relationships) were significantly lower among older PLWHAs when compared to younger PLWHAs (Table 2). Both groups, however, scored nearly the same in environmental quality of life (9.8 versus 10.2; *P* = 0.32) (Table 2). Among older PLWHAs, depression was negatively associated with well-being and quality of life in two domains: physical and psychological health. Subjective well-being was positively related to quality of life in all four domains. Perceived general self-efficacy was not statistically associated with depression, well-being, and quality of life (Table 3).

## 4. Discussion

Although the demographic profile of the HIV epidemic in China is rapidly aging, this is the first study, to our knowledge, to examine psychological and mental health status among older PLWHAs. Our findings show that when compared to younger PLWHAs, older PLWHAs exhibited significantly greater symptoms of depression, poorer well-being, and poorer quality of life in three domains: physical health, psychological health, and social relationships. We also found that a higher level of depression among older PLWHAs was associated with much lower levels of subjective well-being and quality of life (physical health and psychological domain).

Overall participants in our study were found to experience symptoms of clinical depression irrespective of age. This finding is consistent with reports from USA showing depression rates for HIV-positive adults to be twice that of the general population [26]. Older PLWHAs, however, reported greater levels of depression than younger counterparts. As a psychosocial burden, depression likely adds formidable challenges for older PLWHAs to cope with HIV and seek social support. Given these obstacles, it is not surprising that PLWHAs exhibited depressive symptoms in our sample. Moreover, the level of depression reported in this study was much greater among older adults than typically found in research in China on depression unrelated to HIV. For example, in a study of depression among the general Chinese elderly population [25], the mean level of depression measured by the same CES-D scale was between 7.3 and 8.3 and the depression prevalence was between 17 and 22%, compared with the mean level of 10.8−13.6 and prevalence of 48.7–74.2% in our sample. Similarly, the quality of life experienced by PLWHAs in this study also was lower than Chinese general population studies tend to report. For example in a study of Chinese adults, Skevington et al. reported mean scores of 15.8 in physical health, 14.3 in psychological health, 13.7 in social relationships, and 13.2 in the environmental domain [16].

Our data also show that older PLWHAs in this study had poorer quality of life than adult PLWHAs reported in other studies. Shan et al. reported that the physical, psychological, social, and environmental domain scores were 12.9, 12.4, 14.0, and 12.5, respectively, among adult PLWHAs aged between 29 and 60 years old [27]. In addition to the age effects of having a somewhat younger sample, this difference could be due to different stages of disease progression, effects of antiretroviral treatment, or other psychosocial factors. Although AIDS-defining illnesses can be reduced from ART, the likelihood of developing HIV-associated non-AIDS conditions such as cardiovascular disease, lung disease, and cancers can increase with age. The interaction of advancing age, chronic diseases conditions, and psychological problems will continue to affect older PLWHAs' aging process, quality of life, and well-being if no effective intervention exists in this vulnerable population.

In contrast to our expectation, the level of general self-efficacy among older and younger PLWHAs is comparable to the level reported from other studies among Chinese general populations. In a study of 1,003 participants in Hong Kong [28], the mean self-efficacy score was 25.3 among participants aged less than 40 years old, 25.9 among those aged 40–59, and 25.8 among those who were 60 years old and older. In another study, the mean score was 25.5 among Chinese college students [29]. Individuals with adequate self-efficacy are more likely to build resilience to life adversity such as those encountered living with HIV [29].

Despite experiences of significant adversity, individuals with strong resilience may have positive adaptation or coping skills [30]. Resilience as an adaptive process is posited to be important to successful aging [31–33]. The relationships among self-efficacy, resilience, and successful aging are seen as a dynamic process and not a static trait; therefore, it is important to examine the processes by which burdensome and adverse situations may lead to positive adaptations [34]. While resilience has been found to influence quality of life and successful health management among HIV-positive older adults in USA [35], few, if any, empirical studies have longitudinally investigated the dynamic development of resilience and its impact on aging with HIV in China or Asia in general.

Several limitations to this study should be noted. First, due to the nature of a preliminary study with a small sample size, we could not fully investigate and address potential psychosocial factors contributing to depression and quality of life through more complex and in-depth data analysis. Large-scale studies are needed to examine the interaction effects of aging in tandem with psychological and mental health factors among older PLWHAs. Second, the single study site with an intentionally chosen convenience sample may limit the generalizability of our findings. Third, the study relied on self-reported data and consequently may be subject to both recall and social desirability bias. Also, previous studies have found gender differences in reported quality of life in older adults. Due to the small sample size, we could not investigate these differences.

Despite these limitations, our study provides preliminary information regarding the extent to which older PLWHAs in China confront psychological challenges. The findings demonstrate that older PLWHAs reported lower well-being and experienced greater depressive symptoms and poorer quality of life when compared to younger PLWHAs. When coupled with the normal processes of aging and AIDS-associated conditions, the psychosocial and mental health challenges faced by older PLWHAs may go well beyond those of their younger HIV-infected counterparts. Because it was a preliminary study, factors determining the impaired psychological and physical functions and their consequences were not fully investigated. Further research with a larger sample of older PLWHAs is needed to confirm our findings and to understand how aging can accelerate disease progression and affect the psychosocial and mental well-being of older adults living with HIV.

## Figures and Tables

**Table 1:** Demographic characteristics of two subsamples.

	Older PLWHAs∗ number (%)	Younger PLWHAs∗ number (%)	*P* value
Gender			0.53
Female	8 (25.8)	37 (31.6)	
Male	23 (74.2)	80 (68.4)	
Education			0.06
No school or primary school	12 (38.7)	26 (22.2)	
Middle school or above	19 (61.3)	91 (77.8)	
Marital status			0.02
Unmarried	4 (12.9)	40 (34.2)	
Married	27 (87.1)	77 (65.8)	
Years after HIV diagnosis			0.19
<1	15 (48.4)	42 (35.9)	
1-2	12 (38.7)	42 (35.9)	
≥3	4 (12.9)	33 (28.2)	
Being on ART			0.48
Yes	15 (48.4)	65 (55.6)	
No	16 (51.6)	52 (44.4)	

∗People living with HIV/AIDS.

**Table 2:** Distributions of HIV knowledge, self-efficacy, well-being, depression and quality of life.

	Crude mean (SD)			Adjusted mean^2^		
	Older PLWHAs	Younger PLWHAs	*P* value	Older PLWHAs	Younger PLWHAs	*P* value
HIV knowledge^1^	8.5 (3.9)	10.1 (2.3)	0.20	9.3	10.3	0.07
Self-efficacy^1^	24.6 (3.4)	25.3 (3.4)	0.34	23.9	24.6	0.31
Depression	13.6 (5.6)	10.8 (6.5)	0.03	18.6	15.8	0.03
Well-being	7.8 (5.6)	11.7 (6.3)	<0.01	7.6	11.4	<0.01
Quality of life:						
Physical health	10.4 (2.8)	12.2 (2.9)	<0.01	10.3	11.9	<0.01
Psychological^1^	10.1 (2.4)	11.4 (2.8)	0.02	9.5	10.9	0.01
Social relationships	12.0 (2.5)	13.0 (2.6)	0.05	11.8	12.9	0.05
Environment^1^	10.5 (2.7)	10.8 (2.4)	0.41	9.8	10.2	0.32

^1^Logarithmic transformation was applied.

^2^Adjusted for gender, education, and marital status, being on ART, and years after HIV diagnosis.

**Table 3:** Correlations of self-efficacy, depression, well-being, and quality of life among older PLWHAs.

	Depression	Well-being	Physical	Psychological	Social	Environment
Self-efficacy	0.18	−0.28	−0.13	0.16	0.26	0.23
Depression		−0.55**	−0.59**	−0.63**	−0.05	−0.13
Well-being			0.76**	0.68**	0.36*	0.37*
Quality of life:						
Physical health				0.77**	0.35*	0.39*
Psychological					0.42**	0.53**
Social relationships						0.43*
Environment						1.00

*P ≤ 0.05.

**P ≤ 0.01.
